# Diaphragm pacing implantation in Japan for a patient with cervical spinal cord injury: A case report

**DOI:** 10.1097/MD.0000000000029719

**Published:** 2022-06-30

**Authors:** Kazuya Yokota, Muneaki Masuda, Ryuichiro Koga, Masatoshi Uemura, Tadashi Koga, Yasuharu Nakashima, Osamu Kawano, Takeshi Maeda

**Affiliations:** a Department of Orthopaedic Surgery, Japan Labor Health and Welfare Organization Spinal Injuries Center, Fukuoka, Japan; c Department of Rehabilitation Medicine, Japan Labor Health and Welfare Organization Spinal Injuries Center, Fukuoka, Japan; d Department of Surgery, Iizuka Hospital, Fukuoka, Japan; b Departments of Orthopaedic Surgery, Graduate School of Medical Sciences, Kyushu University, Fukuoka, Japan.

**Keywords:** diaphragm pacing stimulation, respiratory dysfunction, spinal cord injury, surgical implantation

## Abstract

Traumatic cervical spinal cord injury (SCI) is a devastating condition leading to respiratory failure that requires permanent mechanical ventilation, which is the main driver of increased medical costs. There is a great demand for establishing therapeutic interventions to treat respiratory dysfunction following severe cervical SCI.

**Patient concerns and diagnosis:**

We present a 24-year-old man who sustained a cervical displaced C2–C3 fracture with SCI due to a traffic accident. As the patient presented with tetraplegia and difficulty in spontaneous breathing following injury, he was immediately intubated and placed on a ventilator with cervical external fixation by halo orthosis. The patient then underwent open reduction and posterior fusion of the cervical spine 3 weeks after injury. Although the patient showed significant motor recovery of the upper and lower limbs over time, only a slight improvement in lung capacity was observed.

**Interventions and outcomes:**

At 1.5 years after injury, a diaphragmatic pacing stimulator was surgically implanted to support the patient’s respiratory function. The mechanical ventilator support was successfully withdrawn from the patient 14 weeks after implantation. We observed that both the vital capacity and tidal volume of the patient were significantly promoted following implantation. The patient finally returned to daily life without any mechanical support.

**Lessons:**

The findings of this report suggest that diaphragmatic pacing implantation could be a promising treatment for improving respiratory function after severe cervical SCI. To our knowledge, this is the first SCI patient treated with a diaphragm pacing implantation covered by official medical insurance in Japan.

**Key Points**

- We present a case of diaphragm pacing implantation for respiratory dysfunction following an upper cervical spinal cord injury.
- Surgical implantation of a diaphragm pacing stimulator allowed the patient to be weaned off the ventilator and return to daily life without any mechanical support.
- After withdrawal from the ventilator, the patient was not reintubated during his long-term follow-up.
- This case represents the first spinal cord injury patient treated with a diaphragm pacing implantation covered by official medical insurance in Japan.

## 1. Introduction

Traumatic spinal cord injury (SCI) is an insult to the central nervous system often resulting in devastating temporary or permanent neurological impairment and disability, such as urinary dysfunction.^[1]^ In particular, patients with severe SCI at the upper cervical level suffer from respiratory problems due to dysfunction of the diaphragm and accessory muscle contraction.^[2]^ The phrenic nerves originate from the C3, C4, and C5 segments of the spinal cord,^[3]^ and approximately 20% of cervical SCIs occur above the C4 level.^[4]^ Neurological status, including motor/sensory and respiratory functions, improves spontaneously over time after incomplete SCI, and the degree of the improvement mainly depends on the initial severity of the injury.^[5–7]^ However, some patients with cervical SCI require permanent mechanical ventilation, including mandatory ventilation or noninvasive positive pressure ventilation, even after their limbs’ function shows a certain degree of recovery.^[8,9]^ Cervical SCI patients with permanent mechanical ventilation have a lifelong risk of respiratory complications such as pneumonia and atelectasis, which are the main drivers of increased medical costs.^[10]^ Since prolonged use of mechanical ventilation may exacerbate morbidity and mortality rates,^[11]^ there is a great demand for establishing therapeutic interventions to treat respiratory dysfunction following severe cervical SCI.

As one of the treatment options for neurogenic respiratory dysfunction, phrenic nerve stimulation by implanting a diaphragm pacing system has been developed over a decade in the United States,^[12,13]^ and several studies have shown that diaphragm pacing supports respiratory function in patients with cervical SCI.^[14,15]^ However, in Japan, there are no reports of diaphragm pacing implantation in patients with cervical SCI. In this case report, we present a patient with SCI at the C2–C3 level suffering from respiratory dysfunction. The patient required ventilator management for more than a year; however, he was able to wean off the ventilator following the implantation of a diaphragm pacing stimulation system.

## 2. Case presentation

**Figure 1.**
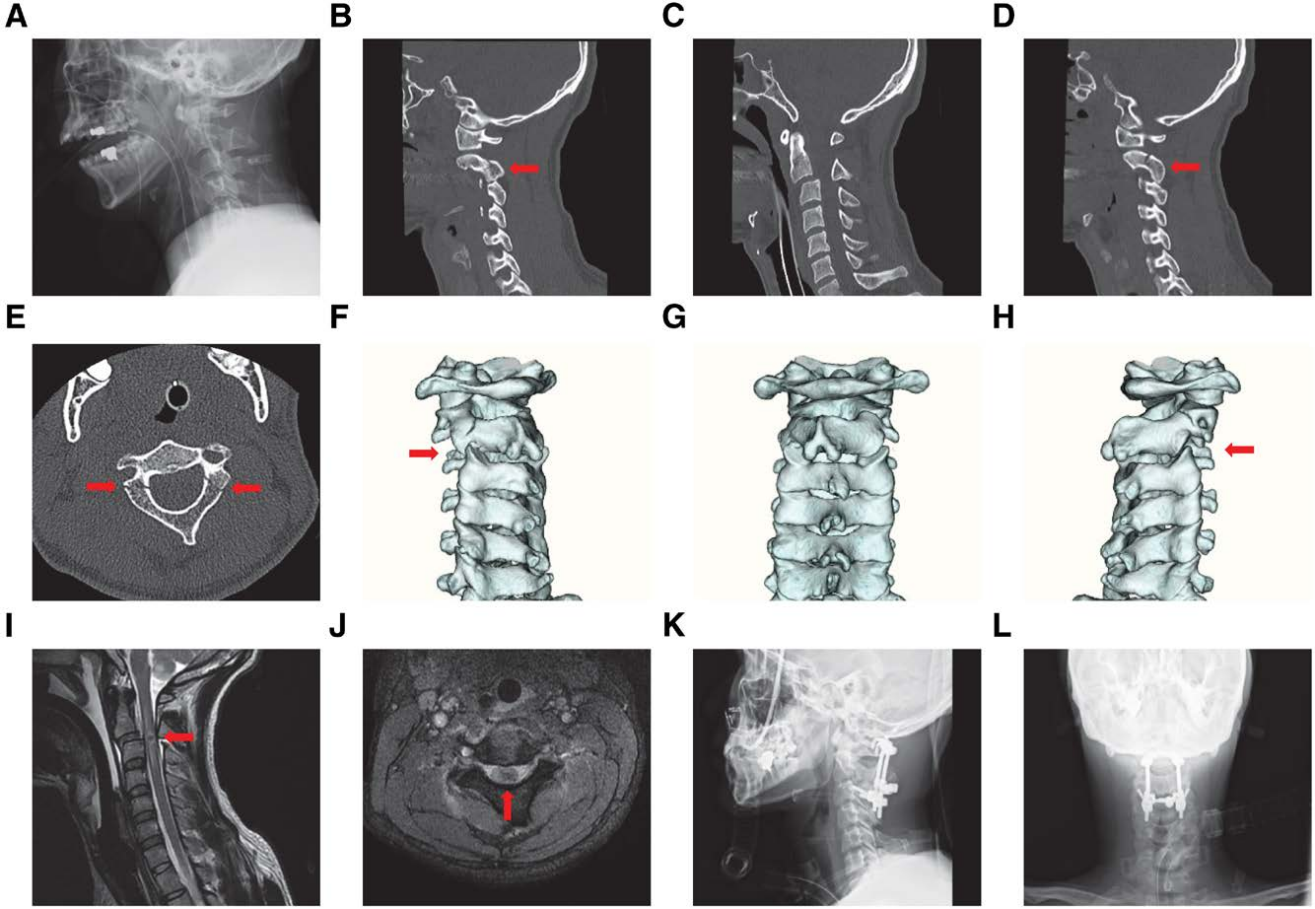
(A) Radiographic image of the patient at the time of injury. The patient suffered from severe tetraplegia with respiratory dysfunction and was intubated immediately after the injury. The image shows the C2 vertebral body displaced anteriorly. Computed tomography images of the cervical spine with C2 dislocated fracture in the sagittal section (B–D), axial section (E), and 3D reconstruction (F–H). Arrow heads show fracture of the left and right pars interarticularis of the axis. The left facet joint at C2–C3 was dislocated, and the superior articular process of C2 was perched on the lateral mass of C3 (B and F), whereas the facet joint at right C2–C3 was subluxated (D and H). MRI T2-weighted images of the patient at the time of injury in the sagittal section (I) and axial section (J) at the C2–C3 level. MRI images show a long segment lesion of T2-weighted hyperintensity signals around the injured level, with T2-weighted hypointensity signals at the epicenter. (K and L) Radiographic images of the patient after posterior cervical fusion 3 wk after the injury. MRI = magnetic resonance imaging.

**Figure 2.**
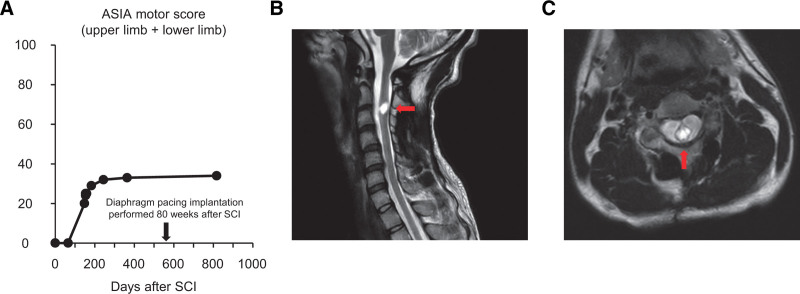
(A) Functional recovery timeline of the patient based on the ASIA motor score (upper limb + lower limb). The motor score reached a plateau at 1-yr postinjury. MRI T2-weighted images of the patient 2 yr after the injury in the sagittal section (B) and axial section (C) at the C2–C3 level. MRI images show syringomyelia with T2-weighted hyperintensity signals at the center of the lesion. ASIA = American Spinal Injury Association, MRI = magnetic resonance imaging, SCI = spinal cord injury.

**Figure 3.**
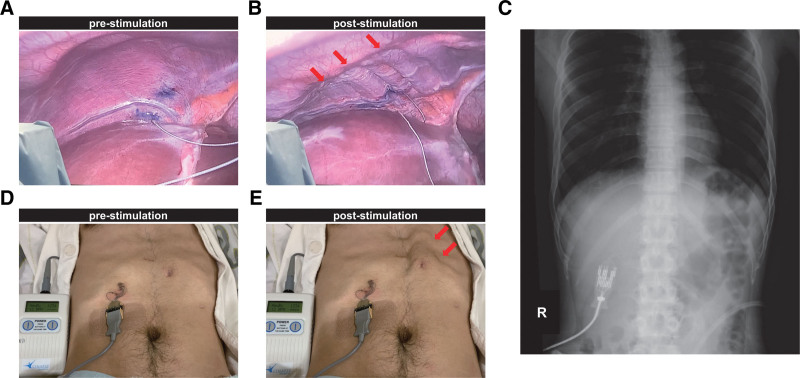
Laparoscopic images before (A) and after (B) diaphragm stimulation. An electrode was placed in the diaphragm at the point where maximal contraction of the diaphragm was identified after the diaphragm was mapped. Arrow heads show the contraction of the diaphragm poststimulation (B). (C) Chest and abdomen radiograph showing phrenic nerve stimulator electrodes connected bilaterally to a pulse generator. Postoperative appearance of the patient before (D) and after (E) diaphragm stimulation. The four implanted electrodes were tunneled to the external pulse generator that stimulated his diaphragm to allow ventilation. In addition to the diaphragm, muscle contraction of the rectus abdominis (arrow heads) can be seen on the exterior poststimulation.

**Figure 4.**
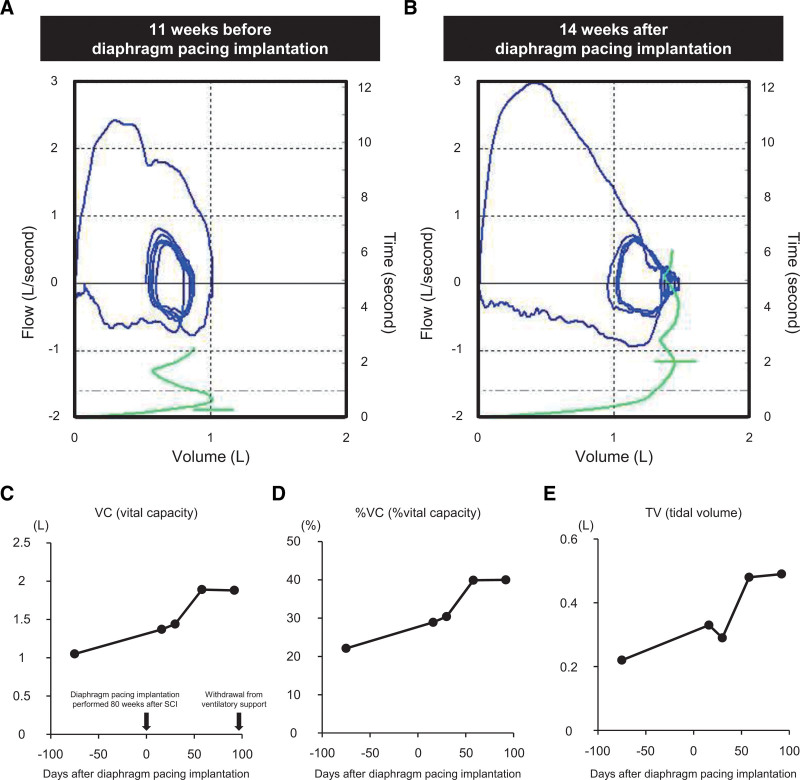
Flow-volume curves of the patient before (A) and after (B) diaphragm pacing implantation. The curves show a significant improvement in the restrictive disorder of the patient after diaphragm pacing implantation. (C–E) The time courses of the significant recovery of respiratory function based on VC, %VC, and TV in the patient are shown. TV = tidal volume, VC = vital capacity.

**Figure 5.**
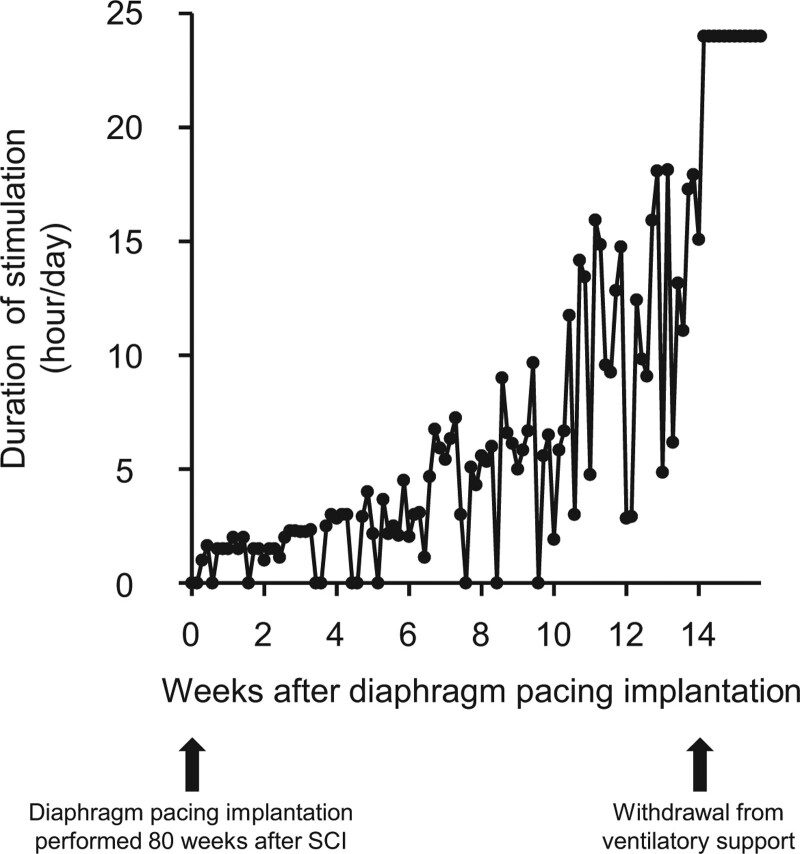
Duration of stimulation after diaphragm pacing implantation. The patient was withdrawn from mechanical support 14 wk after implantation. SCI = spinal cord injury.

A 24-year-old man presented with tetraplegia, respiratory dysfunction, and severely impaired consciousness caused by a traffic accident while working as a member of the Japan Ground Self-Defense Force. He had no history of underlying diseases. Immediately after the patient was transported to the emergency department, he was intubated and placed on a ventilator. Physical examination showed complete flaccid paralysis of both the upper and lower limbs. X-ray and computed tomography scans showed a C2–C3 dislocated cervical injury with fractures at both pars interarticularis of the axis (Hangman’s fracture type II^[16]^). The left facet joint at C2–C3 was dislocated, and the superior articular process of C2 was perched on the lateral mass of C3, whereas the facet joint at right C2–C3 was subluxated (Fig. 1A–H). A magnetic resonance imaging scan of the cervical spine was performed, and the location of the main SCI lesion was detected at the C2–C3 disc level (Fig. 1I and J). Based on the neurological and imaging findings, cervical SCI was diagnosed with American Spinal Injury Association (ASIA) Impairment Scale grade A.^[17]^ Cervical external fixation with a halo vest was performed the next day after the injury. Cervical traction with a force of 2 kg was performed; however, the dislocation was not sufficiently corrected. Seven days after the injury, reduction of the dislocation was successfully performed under general anesthesia using a fluoroscope, and subsequently, a tracheostomy was performed. The patient then underwent posterior cervical fusion 3 weeks after the injury (Fig. 1K and L). At 8 weeks postinjury, as slight spontaneous breathing was observed, he started to wean from the mechanical ventilation and was trained for oral food intake. At 3 months postinjury, his spontaneous breathing was insufficient to maintain gas exchange capacity, such as CO_2_ release without mechanical support, and we estimated that eventual withdrawal of ventilation would be difficult. At 4 months postinjury, slight active movements in the upper and lower limbs were observed, and his muscle strength in both limbs gradually recovered over time after the injury. His ASIA motor score (upper limb + lower limb) 1 year after the injury was 34 points with ASIA Impairment Scale grade C (Fig. 2A). Magnetic resonance imaging images of the cervical spine 2 years after the injury showed a huge cavity in the epicenter at the C2–C3 level, with only a small amount of spinal cord parenchyma remaining (Fig. 2B and C). After confirming in a diaphragmatic nerve stimulation study that stimulation increased contraction in the diaphragm, we decided to perform diaphragmatic pacing implantation in the patient. Diaphragm pacing implantation was performed 1.5 years (80 weeks) after the injury, and intraoperative images of laparoscopy are shown in Fig. 3A and B. Four electrodes were placed on the diaphragm, two on each side, and we confirmed that the diaphragm contracted when stimulated by the electrodes. The four implanted electrodes were tunneled to the external pulse generator that stimulated the diaphragm to allow ventilation (Fig. 3C–E). We performed a respiratory function test before and after diaphragm pacing implantation (Fig. 4A and B), and a significant recovery of respiratory function, such as vital capacity (VC), %VC, and tidal volume after implantation, was observed (Fig. 4C–E). He was withdrawn from mechanical support 14 weeks after implantation (Fig. 5A), and the tracheostomy was completely closed 19 weeks after implantation. After withdrawal from the ventilator, he was not intubated during the 2-year follow-up period.

## 3. Discussion

In this case report, we found that diaphragm pacing implantation significantly improved respiratory function after a cervical SCI. Diaphragmatic pacing not only promoted lung capacity and tidal volume assessed by flow-volume curve but also allowed the patient to be weaned from the ventilator including any artificial support. Further clinical studies investigating diaphragm pacing as a treatment for respiratory dysfunction caused by severe SCI should be conducted.

In a prospective, nonrandomized clinical trial (NCT00420719), the World Federation of Neurology Research Group on Motor Neuron Diseases assessed the effect of diaphragm pacing on patients with amyotrophic lateral sclerosis (ALS) and demonstrated no change in noninvasive ventilation status or ALS functioning respiratory score. On the other hand, its beneficial effects on activities of daily living (ADL), such as sleep state and apnea-hypopneas index, have been reported despite the worsening of the patients’ general condition related to the natural history of such a neurodegenerative disease.^[18–20]^ In Japan, diaphragmatic pacing was also used in a clinical trial to treat respiratory impairment in patients with ALS. However, diaphragmatic pacing did not contribute to prolonging the life prognosis of patients with ALS, and treatment with diaphragmatic pacing was consequently excluded from the insurance coverage.^[21–24]^ Although ALS is a progressive disease,^[25]^ SCI generally causes the most neurological damage immediately after the injury, and the neurological status gradually improves and reaches a plateau during the chronic phase.^[7]^ Given the long-lasting effects of treatment with diaphragmatic pacing in younger patients with SCI,^[14]^ we assumed that diaphragmatic pacing may be more cost-effective in SCI than in ALS. Currently, diaphragmatic pacing is applicable to only two diseases in Japan: SCI and central hypoventilation syndrome.^[26]^ Verification of the therapeutic effect of diaphragmatic pacing has recently started, and to our knowledge, this is the first patient with SCI treated with diaphragm pacing implantation in Japan. Diaphragm pacing was originally intended to be used only as a part-time support for spontaneous breathing; however, surgical implantation of diaphragm pacing consequently allowed the patient to wean off the ventilator in this report. We demonstrated that diaphragmatic pacing contributed not only to the improvement of respiratory function but also to the improvement of ADL, such as respiratory weaning, which will help to establish a therapeutic strategy for SCI in the future.

There were several problems with the NeuRx diaphragmatic pacing system (Synapse Biomedical Inc., Cincinnati, OH) used in this case report.^[23]^ First, the electrodes were connected to the pulse generator outside the body, which could lead to wound infection. Although this patient showed no signs of wound infection during the procedure, close wound observation when placing a diaphragm pacing in a patient with immunocompromised status is necessary.^[27]^ Decubitus ulcers and respiratory tract infections associated with SCI can also trigger wound infection.^[28]^ We believe that if a pulse generator could be implanted inside the body in the same way that a pump is implanted into the body for intrathecal baclofen infusion therapy to treat spasticity,^[29]^ the possibility of infection could be reduced. Second, the conditioning criteria for diaphragmatic stimulation are not yet well established. It is difficult to adjust the stimulation time, intensity, and frequency per day, and changes in the condition settings need to be considered for each patient.^[30]^ If the stimulation time is too long, the diaphragm may not contract sufficiently due to fatigue, and if the stimulation intensity is too strong, the patient may complain of pain in the abdomen. In this study, the stimulation time was started with 30 minutes/d, and the patient was able to receive diaphragmatic stimulation throughout the day (except during meal intake and bathing) 4 months after installation. Further studies determining the setting conditions of electrode stimulation in detail by accumulating cases of cervical SCI are required.

## 4. Conclusion

In this study, we performed diaphragm pacing implantation in a patient with respiratory dysfunction associated with severe cervical SCI. After the implantation, the patient’s respiratory function significantly improved, and he was eventually successfully weaned from the ventilator. Hence, the findings of this study suggest that diaphragm pacing could be a treatment option to improve ADL in patients with respiratory failure following cervical SCI. Further studies confirming its detailed efficacy in clinical practice are warranted.

## Author contributions

Kazuya Yokota designed the study and analyzed the datasets with technical help from Muneaki Masuda. Kazuya Yokota, Ryuichiro Koga, Masatoshi Uemura, and Tadashi Koga collected the patients’ data. Yasuharu Nakashima, Osamu Kawano, and Takeshi Maeda provided experimental support and ideas for the project. Kazuya Yokota supervised the overall project and performed the final manuscript preparation. All authors read and approved the final article.

## Acknowledgments

The authors would like to thank Raymond P. Onders, Department of Surgery, University Hospitals Cleveland Medical Center, for technical support when implanting diaphragm pacing stimulator in the patient.

## References

[1] Ahuja CS Wilson JR Nori S. Traumatic spinal cord injury. Nat Rev Dis Primers 2017;3:17018. 28447605 10.1038/nrdp.2017.18

[2] Malas F Köseoğlu F Kara M. Diaphragm ultrasonography and pulmonary function tests in patients with spinal cord injury. Spinal Cord 2019;57:679–83. 30967603 10.1038/s41393-019-0275-3

[3] Wang C Zhang Y Nicholas T. Neurotization of the phrenic nerve with accessory nerve for high cervical spinal cord injury with respiratory distress: an anatomic study. Turk Neurosurg 2014;24:478–83. 25050670 10.5137/1019-5149.JTN.8335-13.1

[4] Como JJ Sutton ER McCunn M. Characterizing the need for mechanical ventilation following cervical spinal cord injury with neurologic deficit. J Trauma 2005;59:912–6. 16374281 10.1097/01.ta.0000187660.03742.a6

[5] Randelman M Zholudeva LV Vinit S. Respiratory training and plasticity after cervical spinal cord injury. Front Cell Neurosci 2021;15:700821. 34621156 10.3389/fncel.2021.700821 PMC8490715

[6] Michel-Flutot P Mansart A Deramaudt TB. Permanent diaphragmatic deficits and spontaneous respiratory plasticity in a mouse model of incomplete cervical spinal cord injury. Respir Physiol Neurobiol 2021;284:103568. 33144274 10.1016/j.resp.2020.103568

[7] Fawcett JW Curt A Steeves JD. Guidelines for the conduct of clinical trials for spinal cord injury as developed by the ICCP panel: spontaneous recovery after spinal cord injury and statistical power needed for therapeutic clinical trials. Spinal Cord 2007;45:190–205. 17179973 10.1038/sj.sc.3102007

[8] Lertudomphonwanit T Wattanaapisit T Chavasiri C. Risk factors relating to the need for mechanical ventilation in isolated cervical spinal cord injury patients. J Med Assoc Thai 2014;97(Suppl 9):S10–5. 25365883

[9] Kornblith LZ Kutcher ME Callcut RA. Mechanical ventilation weaning and extubation after spinal cord injury: a Western Trauma Association multicenter study. J Trauma Acute Care Surg 2013;75:1060–9. 24256682 10.1097/TA.0b013e3182a74a5b PMC3837348

[10] Winslow C Bode RK Felton D. Impact of respiratory complications on length of stay and hospital costs in acute cervical spine injury. Chest 2002;121:1548–54. 12006442 10.1378/chest.121.5.1548

[11] McGrath R Hall O Peterson M. The association between the etiology of a spinal cord injury and time to mortality in the United States: a 44-year investigation. J Spinal Cord Med 2019;42:444–52. 30124389 10.1080/10790268.2018.1505311 PMC6718184

[12] Onders RP Elmo MJ Ignagni AR. Diaphragm pacing stimulation system for tetraplegia in individuals injured during childhood or adolescence. J Spinal Cord Med 2007;30(Suppl 1):S25–9. 17874683 10.1080/10790268.2007.11753965 PMC2031991

[13] Onders RP Dimarco AF Ignagni AR. Mapping the phrenic nerve motor point: the key to a successful laparoscopic diaphragm pacing system in the first human series. Surgery 2004;136:819–26. 15467667 10.1016/j.surg.2004.06.030

[14] Onders RP Elmo M Kaplan C. Long-term experience with diaphragm pacing for traumatic spinal cord injury: early implantation should be considered. Surgery 2018;164:705–11. 30195400 10.1016/j.surg.2018.06.050

[15] Posluszny JA Jr. Onders R Kerwin AJ. Multicenter review of diaphragm pacing in spinal cord injury: successful not only in weaning from ventilators but also in bridging to independent respiration. J Trauma Acute Care Surg 2014;76:303–9. 24458038 10.1097/TA.0000000000000112

[16] Schneider RC Livingston KE Cave AJ. “Hangman’s fracture” of the cervical spine. J Neurosurg 1965;22:141–54. 14288425 10.3171/jns.1965.22.2.0141

[17] Rupp R Biering-Sørensen F Burns SP. International standards for neurological classification of spinal cord injury: revised 2019. Top Spinal Cord Inj Rehabil 2021;27:1–22. 10.46292/sci2702-1 PMC8152171 34108832

[18] Amirjani N Kiernan MC McKenzie DK. Is there a case for diaphragm pacing for amyotrophic lateral sclerosis patients? Amyotroph Lateral Scler 2012;13:521–7. 22632380 10.3109/17482968.2012.673169

[19] Gonzalez-Bermejo J Morélot-Panzini C Salachas F. Diaphragm pacing improves sleep in patients with amyotrophic lateral sclerosis. Amyotroph Lateral Scler 2012;13:44–54. 22023158 10.3109/17482968.2011.597862

[20] Onders RP Elmo M Kaplan C. Final analysis of the pilot trial of diaphragm pacing in amyotrophic lateral sclerosis with long-term follow-up: diaphragm pacing positively affects diaphragm respiration. Am J Surg 2014;207:393–7. 24439161 10.1016/j.amjsurg.2013.08.039

[21] Le Pimpec-Barthes F Legras A Arame A. Diaphragm pacing: the state of the art. J Thorac Dis 2016;8(Suppl 4):S376–86. 27195135 10.21037/jtd.2016.03.97 PMC4856845

[22] McDermott CJ Bradburn MJ Maguire C. DiPALS: diaphragm pacing in patients with amyotrophic lateral sclerosis - a randomised controlled trial. Health Technol Assess 2016;20:1–186. 10.3310/hta20450 PMC4939419 27353839

[23] DiPALS Writing Committee; DiPALS Study Group Collaborators. Safety and efficacy of diaphragm pacing in patients with respiratory insufficiency due to amyotrophic lateral sclerosis (DiPALS): a multicentre, open-label, randomised controlled trial. Lancet Neurol 2015;14:883–92. 26234554 10.1016/S1474-4422(15)00152-0

[24] Woo A Tchoe HJ Shin HW. Assisted breathing with a diaphragm pacing system: a systematic review. Yonsei Med J 2020;61:1024–33. 33251776 10.3349/ymj.2020.61.12.1024 PMC7700882

[25] Ito H Kamei T Odake S. An autopsy case of amyotrophic lateral sclerosis with diaphragm pacing. Intern Med 2016;55:3511–3. 27904119 10.2169/internalmedicine.55.7130 PMC5216153

[26] Gonzalez-Bermejo J C LL Similowski T. Respiratory neuromodulation in patients with neurological pathologies: for whom and how? Ann Phys Rehabil Med 2015;58:238–44. 26260006 10.1016/j.rehab.2015.07.001

[27] Kopp MA Watzlawick R Martus P. Long-term functional outcome in patients with acquired infections after acute spinal cord injury. Neurology 2017;88:892–900. 28130472 10.1212/WNL.0000000000003652 PMC5331871

[28] Dinh A Bouchand F Davido B. Management of established pressure ulcer infections in spinal cord injury patients. Med Mal Infect 2019;49:9–16. 29937316 10.1016/j.medmal.2018.05.004

[29] Khurana SR Garg DS. Spasticity and the use of intrathecal baclofen in patients with spinal cord injury. Phys Med Rehabil Clin N Am 2014;25:655–69. 25064793 10.1016/j.pmr.2014.04.008

[30] Cavka K Fuller DD Tonuzi G. Diaphragm pacing and a model for respiratory rehabilitation after spinal cord injury. J Neurol Phys Ther 2021;45:235–42. 34049339 10.1097/NPT.0000000000000360 PMC8711094

